# Large scale cytokine profiling uncovers elevated IL12-p70 and IL-17A in severe pediatric acute respiratory distress syndrome

**DOI:** 10.1038/s41598-021-93705-8

**Published:** 2021-07-08

**Authors:** Judith Ju Ming Wong, Herng Lee Tan, Jieliang Zhou, Jan Hau Lee, Jing Yao Leong, Joo Guan Yeo, Yie Hou Lee

**Affiliations:** 1 Children’s Intensive Care Unit, Department of Pediatric Subspecialities, KK Women’s and Children’s Hospital, 100 Bukit Timah Road, 229899 Singapore, Singapore; 2 Translational Immunology Institute, Singhealth Duke-NUS, 20 College Road, Singapore, 169856 Singapore; 3 Duke-NUS Medical School, 8 College Road, Singapore, 169857 Singapore; 4 KK Research Centre, KK Women’s and Children’s Hospital, 100 Bukit Timah Road, Singapore, 229899 Singapore

**Keywords:** Cytokines, Infectious diseases, Biomarkers, Paediatric research

## Abstract

The specific cytokines that regulate pediatric acute respiratory distress syndrome (PARDS) pathophysiology remains unclear. Here, we evaluated the respiratory cytokine profile in PARDS to identify the molecular signatures associated with severe disease. A multiplex suspension immunoassay was used to profile 45 cytokines, chemokines and growth factors. Cytokine concentrations were compared between severe and non-severe PARDS, and correlated with oxygenation index (OI). Partial least squares regression modelling and regression coefficient plots were used to identify a composite of key mediators that differentially segregated severe from non-severe disease. The mean (standard deviation) age and OI of this cohort was 5.2 (4.9) years and 17.8 (11.3), respectively. Early PARDS patients with severe disease exhibited a cytokine signature that was up-regulated for IL-12p70, IL-17A, MCP-1, IL-4, IL-1β, IL-6, MIP-1β, SCF, EGF and HGF. In particular, pro-inflammatory cytokines (IL-6, MCP-1, IP-10, IL-17A, IL-12p70) positively correlated with OI early in the disease. Whereas late PARDS was characterized by a differential lung cytokine signature consisting of both up-regulated (IL-8, IL-12p70, VEGF-D, IL-4, GM-CSF) and down-regulated (IL-1β, EGF, Eotaxin, IL-1RA, and PDGF-BB) profiles segregating non-severe and severe groups. This cytokine signature was associated with increased transcription, T cell activation and proliferation as well as activation of mitogen-activated protein kinase pathway that underpin PARDS severity.

## Introduction

Pediatric acute respiratory distress syndrome (PARDS) accounts for less than 5% of pediatric intensive care unit (PICU) admissions but is disproportionately associated with high mortality and morbidity^1,2^. PARDS is clinically diagnosed^3^ and arises mainly from respiratory infections^2^. Development of PARDS is unpredictable and the mainstay of treatment has thus far, been supportive^2,4^. Comprehensive translational studies in PARDS to identify effective therapies have been limited, specifically, the interrogation of biological processes occurring in the pulmonary microenvironment has been constrained by (a) the relatively low incidence, (b) under recognition and (c) difficulty of obtaining bronchoalveolar lavage in children^5^.

PARDS, like its adult counterpart, acute respiratory distress syndrome (ARDS), is associated with intense lung inflammation, edema and proteinaceous alveolar exudation resulting in impaired oxygenation^6^. Release of proinflammatory cytokines from injured lung architecture (alveolar and bronchial cells) and immune cells weakens the capillary and alveolar endothelium^7,8^. As damage progresses, proteins leak into the alveolar space, further stimulating the influx of neutrophils and macrophages into the area, propagating the inflammatory response that ultimately leads to diffuse alveolar damage^7,9^. The resultant hypoxemia is secondary to severe ventilation–perfusion mismatch.

Cytokines in ARDS have been extensively studied in the adult population in the hope of identifying potential biomarkers and therapeutic targets^10–12^. However, extrapolation of data gleaned from adults to the pediatric population is inappropriate because of differing clinical definitions^3,13^, epidemiology^2,14^ developmental pulmonary mechanical, microstructural and immunological factors^15^. Few studies have been conducted to describe plasma levels of cytokines in children with PARDS. Plasma IL-6 levels were up-regulated in children with severe PARDS in the first week of disease^16^. Elevations of plasma pro-inflammatory (IL-6, IL-8, IL-18) and anti-inflammatory cytokines (IL-10 and TNF-R2) in non-survivors compared to survivors was demonstrated in a large PARDS multicentre study involving 194 children with heterogenous case histories^17^. These cytokines were found to correlate with both the oxygenation index (OI) and Pediatric Risk of Mortality 3 (PRISM-3) score^17^. One smaller study examined lower respiratory tract cytokines in children intubated for respiratory failure secondary to respiratory infection and demonstrated that CCL7 was found to correlate with severity of illness^18^. In this study, only three patients fulfilled criteria for moderate/severe PARDS.

We postulate that evaluating the cytokine response in the pulmonary microenvironment of patients with PARDS will be more informative than the systemic compartment, especially in the context of direct lung injury. As such, our study aimed to evaluate the cytokine profile in the deep tracheal lavage fluid (DTL) (surrogate for bronchoalveolar lavage fluid)^19^ and plasma in children with PARDS to determine the profile associated with severe disease. We hypothesized that the pulmonary cytokine profile is more closely associated with an inflammatory signature that correlates with OI, a clinical marker of pulmonary disease severity, as opposed to clinical markers of systemic disease severity. We will also explored the trend in cytokine concentrations at early and late phases of PARDS to determine if the mediators for continual severe disease changed temporally with disease course.

## Results

Over the 1-year study period, there were 45 patients who fulfilled the Pediatric Acute Lung Injury Consensus Conference (PALICC) criteria for PARDS^3^. Of these, 16 patients were selected who had evidence of direct lung injury and sufficient blood and DTL fluid samples for analysis (Table 1). All patients had pneumonia, and 12 patients also fulfilled criteria for sepsis. The mean (standard deviation) age and OI of this cohort was 5.2 (4.9) years and 17.8 (11.3), respectively. Notably, all patients in the non-severe group had a positive polymerase chain reaction (PCR) test for common respiratory viruses compared to the severe group which only had 3/8 (37.5%) positive viral PCR. Severe PARDS was expectedly associated with greater use of transfusions, oscillatory ventilation, pulmonary vasodilators, systemic corticosteroids, neuromuscular blockers and extracorporeal membrane oxygenation – though this was only statistically significant for transfusions. The overall PICU mortality in this cohort was 2/16 (12.5%), and both non-survivors had severe PARDS.

**Table 1:** Clinical characteristics of patients in the non-severe and severe groups.

Characteristics	Non-severe (n = 8)	Severe (n = 8)	P value
Age	3.0 (2.7)	7.4 (5.7)	0.069
Male gender	5 (62.5)	5 (62.5)	1.000
Chronic comorbidities	7 (87.5)	6 (75)	1.000
Oxygenation index	10.6 (3.6)	23.1 (12.2)	**0.033**
PIM 2 score	10.5 (12.1)	25.5 (30.0)	0.210
PELOD score	6.4 (7.3)	15.8 (14.2)	0.154
**Infective etiology**			
Viral	8 (100)	3 (37.5)	**0.026**
Bacterial	5 (52.5)	3 (37.5)	0.620
Co-infection	5 (62.5)	1 (12.5)	0.119
No organism	0 (0)	3 (37.5)	0.200
Inotrope	5 (62.5)	7 (75)	0.569
Transfusion	1 (12.5)	7 (87.5)	**0.010**
HFOV	2 (25.0)	4 (50.0)	0.608
Pulmonary vasodilators	0 (0)	3 (37.5)	0.200
Prone	4 (50)	2 (25)	0.608
Steroids	5 (62.5)	7 (75)	0.569
NMB	1 (12.5)	5 (62.5)	0.119
Diuretics	8 (100)	7 (87.5)	1.000
ECMO	0 (0)	2 (25)	0.467
Multiorgan dysfunction	5 (62.5)	8 (100)	0.200
Mortality	0 (0)	2 (25)	0.467
VFD	17.3 (10.4)	10.5 (11.0)	0.228
IFD	14.1 (8.2)	8.8 (9.8)	0.279

Bold values signify *p* < 0.05.

Categorical and continuous data are presented as counts (percentages) and mean (standard deviation) respectively.

*PIM 2* Pediatric Index of Mortality, *PELOD* Pediatric Logistic Organ Dysfunction, *HFOV* high frequency oscillatory ventilation, *NMB* neuromuscular blockade, *ECMO* extracorporeal membrane oxygenation, *VFD* ventilator free days, *IFD* intensive care unit free days.

### Deep tracheal lavage fluid cytokines in early PARDS (timepoint 1)

**Figure 1:**
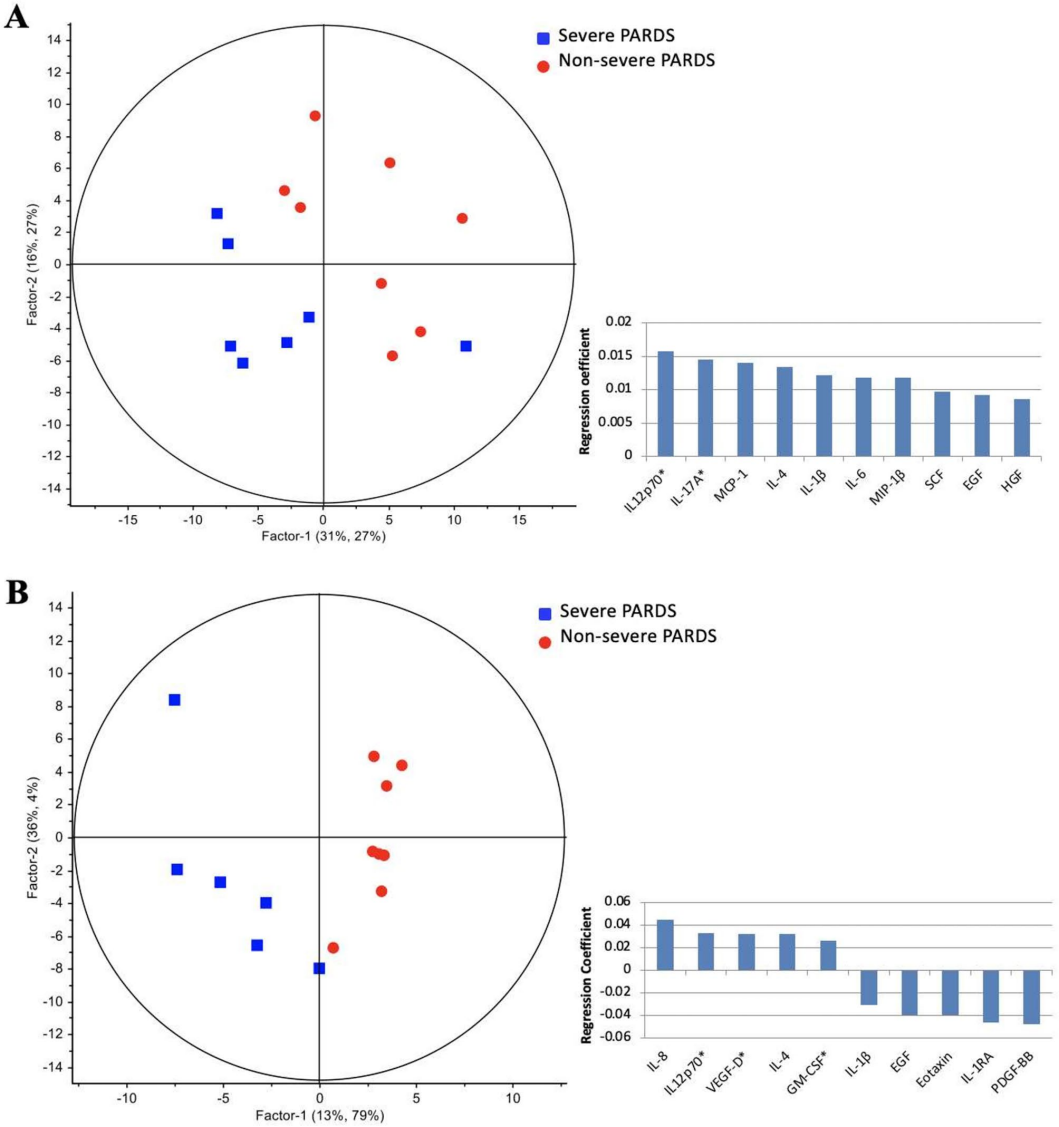
Respiratory cytokines showed distinct segregation between non-severe and severe PARDS in the partial least squares regression model (left) in early (**A**) and late (**B**) timepoints of disease. Regression coefficient plots identifying the top ten respiratory cytokines associated with severe PARDS (right). *Raised on both univariate and multivariate analysis.

The concentration levels of 45 DTL cytokines quantified using multiplex suspension immunoassays comparing early and late timepoints were similar (*p* > 0.05; Supplementary Table 1A) regardless of severity. When comparing the DTL cytokines of severe vs. non-severe cases in the early timepoint, multivariate analysis using partial least squares regression (PLSR) demonstrated clear segregation of clusters corresponding to the comparison groups based on their cytokine profiles (principal component 1: 47%, principal component 2: 54%) (Fig. 1A). A 10-cytokine signature comprising of up-regulated IL-12p70, IL-17A, MCP-1, IL-4, IL-1β, IL-6, MIP-1β, SCF, EGF and HGF distinguished severe from non-severe PARDS. Pathway enrichment analysis of the 10-cytokine signature revealed positive regulation of transcription, T cell proliferation/activation and activation of mitogen-activated protein kinase (MAPK) as enriched Gene Ontology (GO) biological processes (Supplementary Table 2). Using the upregulated cytokine data, unbiased bioinformatic analysis also identified several associated respiratory diseases from the Gene Association Disease (GAD) database.

**Figure 2:**
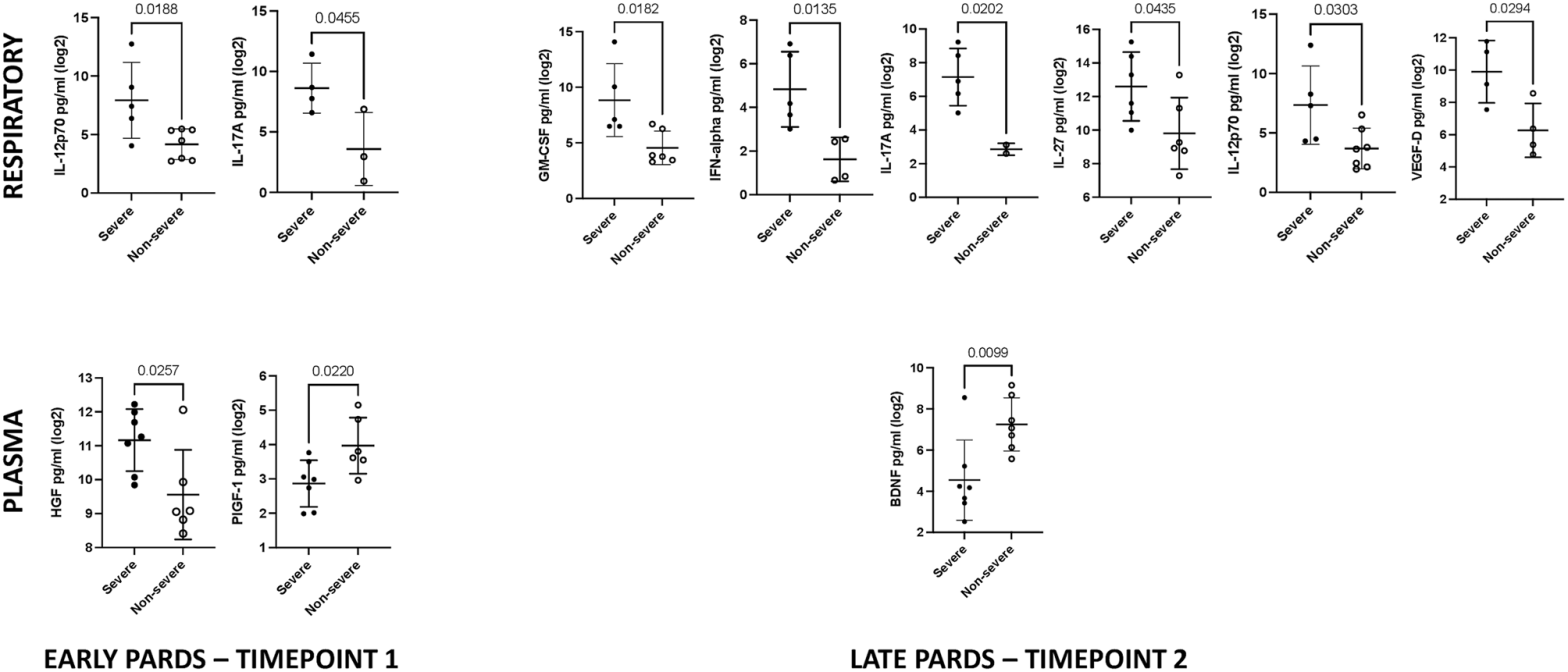
Respiratory and plasma cytokine concentration in severe and non-severe PARDS at timepoint 1 and 2. *p*-value indicates the difference between the two severity groups using t-test.

**Figure 3:**
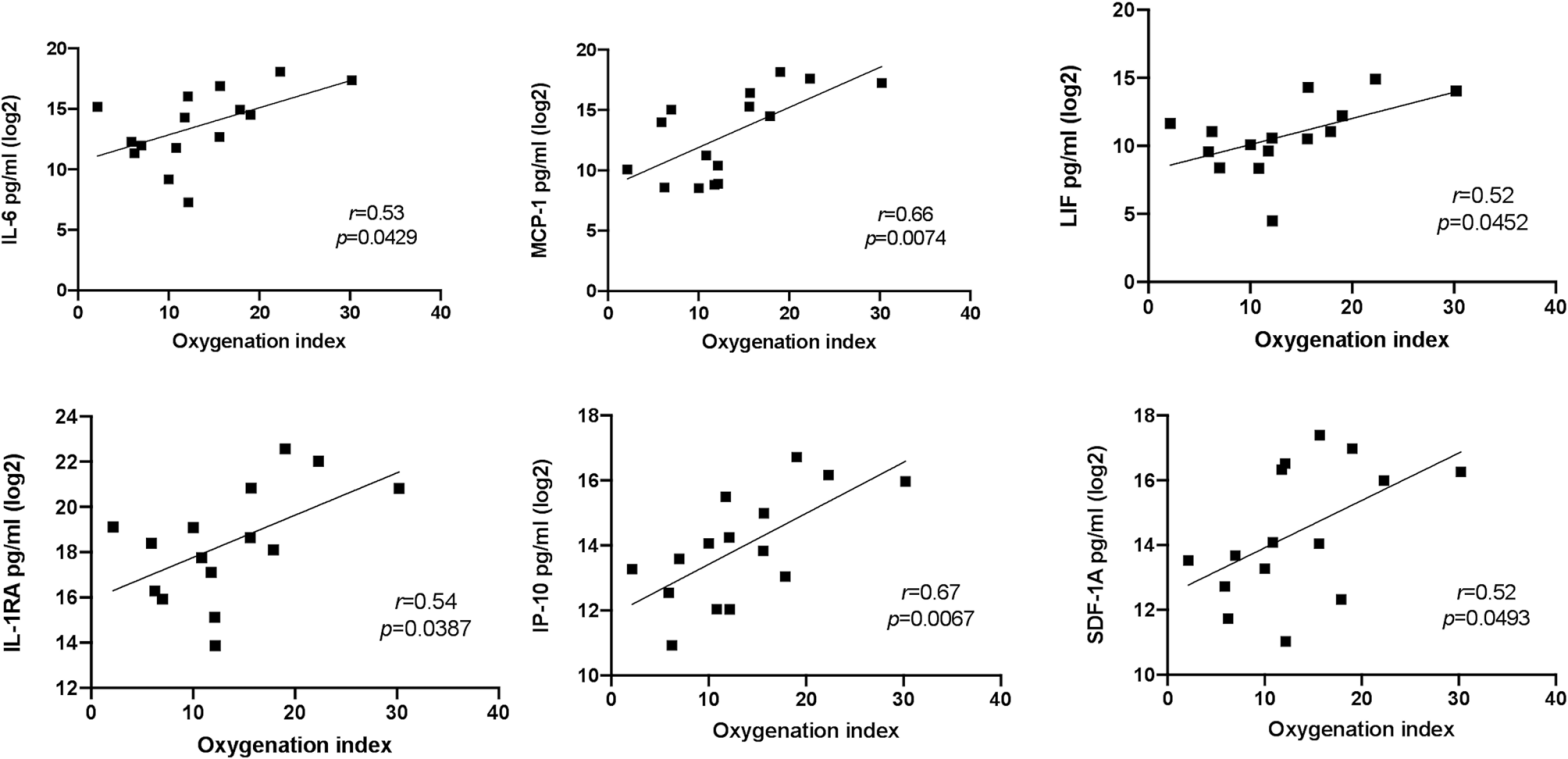
Correlation plots between early timepoint respiratory cytokines and the oxygenation index. *r* = Pearson’s correlation coefficient.

Involvement of pro-inflammatory cytokines IL-17A and IL-12p70 (*p* = 0.0455 and *p* = 0.0188 respectively) in the early timepoint was identified by univariate analysis (Fig. 2), consistent with the PLSR analysis. IL-17A [8.6 (2.1) vs. 3.6 (3.0)] and IL-12p70 [7.9 (3.2) vs. 4.2 (1.3)] were higher in the severe compared to the non-severe group (Supplementary Table 3A). DTL cytokines which correlated significantly with the degree of lung disease (OI) included IL-1RA (*r* = 0.54), IL-6 (*r* = 0.53), IP-10 (*r* = 0.67), LIF (*r* = 0.52), MCP-1 (*r* = 0.66) and SDF-1α (*r* = 0.52) (Fig. 3). None of the DTL cytokines correlated with the systemic severity of illness scores [Pediatric Index of Mortality 2 (PIM 2)^20^ and Pediatric Logistic Organ Dysfunction (PELOD)^21^ scores.) (Supplementary Table 4).

### Deep tracheal lavage fluid cytokines in late PARDS (timepoint 2)

In the late timepoint, widespread changes in DTL cytokines segregated severe from non-severe cases to a greater extent (principal component 1: 49%, principal component 2: 83%) (Fig. 1B). Comparing severe to non-severe PARDS at this second timepoint, a cytokine signature comprising of up-regulated and down-regulated cytokines (IL-8, IL-12p70, VEGF-D, IL-4, GM-CSF, IL-1β, EGF, Eotaxin, IL-1RA, and PDGF-BB) was uncovered. Notably, IL-12p70 remains elevated in severe cases in the late timepoint.

In the univariate analysis, GM-CSF [8.8 (3.3) vs. 4.6 (1.5); *p* = 0.0182], IFN-α [4.8 (1.7) vs. 1.6 (1.0); *p* = 0.0135], IL-17A [7.1 (1.7) vs. 2.9 (0.4); *p* = 0.0202], IL-27 [12.6 (2.0) vs. 9.8 (2.1); *p* = 0.0435], IL-12p70 [7.3 (3.3) vs. 3.7 (1.7); *p* = 0.0303] and VEGF-D [9.9 (1.9) vs. 6.3 (1.7); *p* = 0.0294] were higher in the severe compared to non-severe group (Fig. 2 and Supplementary Table 5A). None of the DTL cytokines correlated with the OI, nor systemic severity of illness scores (PIM 2 and PELOD scores) (Supplementary Table 4). Collectively, the data suggested an early pro-inflammatory cytokine response that underpins PARDS severity, of which IL-12p70 and IL-17A remains elevated in the late phase of severe cases.

### Plasma cytokines in early PARDS (timepoint 1)

**Figure 4:**
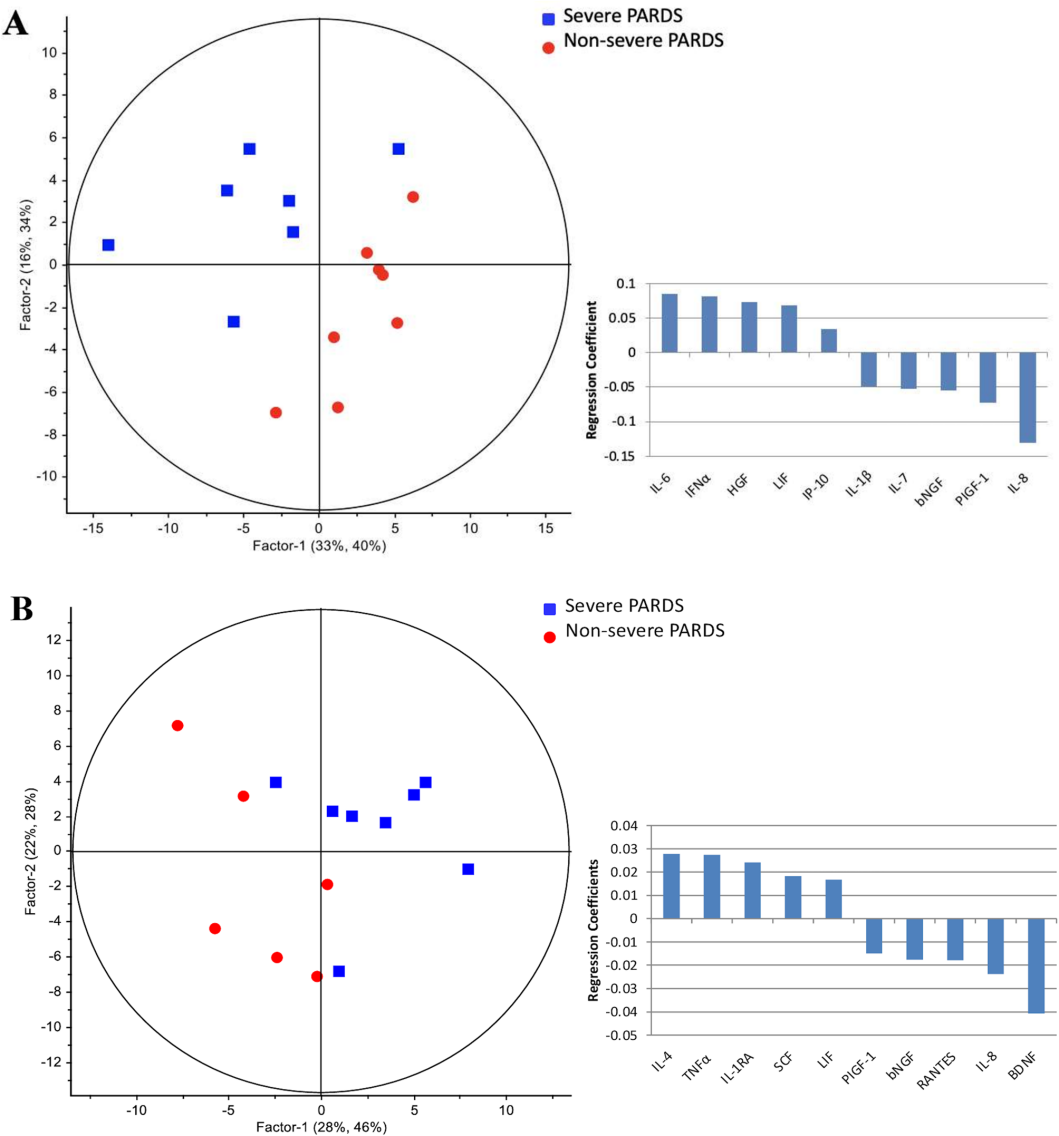
Plasma cytokines showed distinct segregation between non-severe and severe PARDS in the partial least squares regression model (left) in early (**A**) and late (**B**) timepoints of disease. Regression coefficient plots identifying the top ten plasma cytokines associated with severe PARDS (right).

The PLSR result for plasma cytokines is presented in Fig. 4A showing cytokine profiles which clearly segregated non-severe and severe groups into two clusters with up-regulation of IL-6, IFN-alpha, HGF, LIF and IP-10, and down-regulation of IL-1β, IL-7, bNGF, PIGF-1 and IL-8.

In the univariate analysis, only HGF [11.2 (0.9) vs. 8.6 (1.3); *p* = 0.0257] was higher in the severe compared to the non-severe group (Fig. 2 and Supplementary Table 3B). Whereas, PIGF-1 [2.9 (0.7) vs. 4.0 (0.8); *p* = 0.0220] was lower in the severe compared to the non-severe group (Fig. 2). Several plasma cytokines correlated significantly with the OI, PIM and PELOD scores. IL-6 (*r* = 0.66), IL-10 (*r* = 0.58) and VEGF-A (*r* = 0.60) were positively correlated with the OI (Supplementary Fig. 1). MCP-1 (*r* = − 0.58) and SCF (*r* = 0.54) correlated with the PIM 2 score, and IL-27 (*r* = 0.60), IL-15 (*r* = − 0.63) and HGF (*r* = 0.77) with the PELOD score.

### Plasma cytokines in late PARDS (timepoint 2)

The PLSR plots comparing non-severe and severe disease during the course of PARDS also achieved good separation (Fig. 4B) and identified a plasma cytokine signature associated with up-regulation of IL-4, TNF-α, IL-1RA, SCF and LIF, and down-regulation of PIGF-1, bNGF, RANTES, IL-8 and BDNF.

In the univariate analysis, only BDNF [4.5 (1.9) vs. 7.2 (1.3); *p* = 0.0099] was lower in the severe compared to non-severe group at resolution (Fig. 2 and Supplementary Table 5B). Only HGF (*r* = 0.76) correlated with PELOD score (Supplementary Table 4). Similar to DTL fluid, plasma cytokine concentrations were indifferent between the early and late timepoints, except IL-8 which showed a significant decrease between at the second timepoint [8.8 (0.0) vs. 5.2 (1.3); *p* = 0.0185] (supplementary Table 1B).

## Discussion

**Figure 5:**
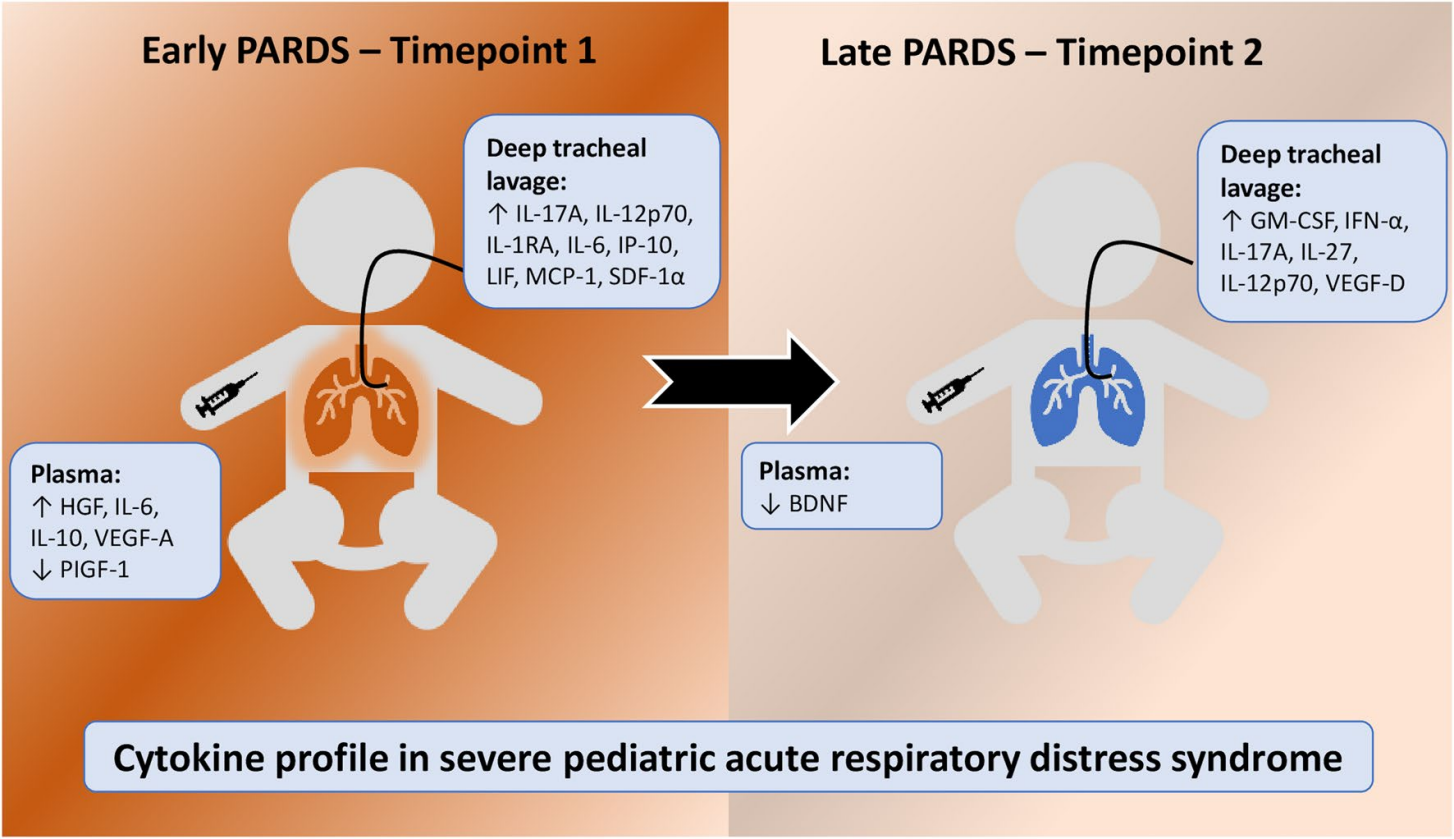
Cytokine profile in severe pediatric acute respiratory distress syndrome. *PARDS* pediatric acute respiratory distress syndrome.

In this cohort of pneumonia induced PARDS, several respiratory cytokines were found to be distinctively associated with lung disease. Some inflammatory drivers (IL-17A, IL-12p70, IL-6, MCP-1, IP-10) were found to be higher in the lungs or correlated with severe PARDS early in the disease, whereas, others were found to peak later in the disease (GM-CSF, IFN-α). Both univariate and multivariate analysis indicated IL-12p70 and IL-17A as an early duplex cytokine signature. Varying immunoregulatory, homeostatic and repair factors (IL-1RA, LIF, SDF-1α, IL-27, IL-10, HGF, VEGF-A, VEGF-D) in the lungs and plasma were also observed to be higher or correlated with severe lung disease during the course of illness, in line with the knowledge that cytokines form biological networks that manifests biochemically and physiologically^8,22^. These are summarised in Fig. 5.

Our study reports a higher production of IL-12p70 in severe compared with non-severe PARDS at both early [7.9 (3.2) vs. 4.2 (1.3); *p* = 0.0188] and late [7.3 (3.3) vs. 3.7 (1.7); *p* = 0.0303] phases of disease. Respiratory concentrations of IL-12p70 were also evidently higher than plasma concentrations [5.5 (2.9) vs. 3.4 (1.5); *p* = 0.0015). IL-12p70 is commonly expressed by antigen presenting cells^23^. Part of the IL-12 cytokine family, IL-12p70 consists of subunits p35 and p40, and its main function includes induction of IFNγ-production from T cells, enhancement of cytotoxic T cells (CTL), and differentiation of naïve T cells into Th-1 effectors, suggesting a key role for IL-12 in the development of cell-mediated immunity (CMI)^23,24^. IL-27 also being part of the IL-12 family, plays a dual pro-inflammatory and immunoregulatory role^25^. IL-27 is involved in initial Th-1 progression via the STAT1-mediated T-box expressed in T cells (T-bet) pathway, but later induction of IL-10 suppresses Th-1, Th-2 and Th-17 responses^26^. It showed a higher expression in severe PARDS which was apparent at later stages of the disease, likely related to the need to attenuate uncontrolled pulmonary inflammation.

Another cytokine found to be associated with severe PARDS, IL-17, is secreted by Th-17, γδ T cells, invariant natural killer cells (iNKT), and type three innate lymphoid cells (ILC3) which drives neutrophil infiltration and pathogen clearance^27,28^. It binds to IL-17 receptors which consist of heterodimeric complexes^29^ and are expressed on a variety of cells, including epithelial cells and neutrophils. Its binding results in activation of downstream signalling for neutrophil chemotaxis, including IL-8, MCP-1 and IP-10, and activating factors IL-6 and GM-CSF, which were also consistently found to be raised in our study^30,31^. Enhanced production of MCP-1 and IP-10 in the lungs of patients with PARDS likely resulted in increased recruitment of circulating neutrophils, monocytes and macrophages in keeping with Th-1 responses^32,33^. Whereas, GM-CSF exert anti-apoptotic properties on neutrophils causing accumulation of neutrophils in the lungs^34^.

Early expression of IL-6 showed an increase corresponding to the degree of lung injury within 24 h of PARDS in the lung microenvironment (*r* = 0.53), as well as, systemically (*r* = 0.66). IL-6 concentrations were significantly higher in the respiratory samples [13.4 (2.8) vs. 6.4 (2.6); *p* < 0.001] and is consistent with clinical evidence of direct lung injury. IL-6 is produced by the innate immune system in response to activation of pathogen associated molecular patterns (PAMPs) or damage associated molecular patterns (DAMPs)^8^.The lack of reduction in IL-6 with disease resolution indicates that potential involvement of other mediators in disease resolution and the need to evaluate the lung inflammatory microenvironment in a holistic and relational manner. Leukemia inhibitory factor (LIF) is also a member of the IL-6 family of cytokines, and has been shown to be induced by IL-1β in pneumonia^35^. Lung epithelium including type 2 alveolar cells, and to a lesser extent, alveolar macrophages, are the main source of LIF^36^. LIF activates STAT3 signalling and upregulates phospholipase A2^37^. Its presence was shown to be important in controlling lung injury, achieving tissue homeostasis after pneumonia and correlated with severity of lung injury in patients with ARDS^38,39^. Our study identified the presence of LIF in pneumonia induced PARDS and showed a weak correlation with severity of lung disease (*r* = 0.52).

Our study has several limitations. Firstly, the sample size is small and a larger cohort will be necessary to validate its findings. Moreover, future inclusion of healthy controls will provide useful normative data. Secondly, though deep tracheal lavage was used as a surrogate for bronchoalveolar lavage specimens, there is no data which confirms they are directly comparable and representative of alveolar epithelial lining fluid. Lastly, confirmation of mechanistic and functional consequences of the identified signatures will require functional assays and animal testing. These limitations will be the basis for further experimental steps in identifying key biomarkers/signatures in the pathogenesis of PARDS.

## Conclusion

The immunological processes occurring in the lungs and systemic circulation of children with PARDS are poorly studied. Our study identifies a complex mixture of pro-inflammatory cytokines (IL-6, MCP-1, IP-10, IL-17A, IL-12p70, GM-CSF, IFN-α), immunoregulatory cytokines (IL-12p70, IL-1RA, IL-27, IL-10) and homeostatic/repair factors (PIGF-1, HGF, VEGF-A, VEGF-D) which were associated with disease severity and time course of PARDS. An early duplex cytokine signature involving IL-12p70 and IL-17A was identified to underpin PARDS severity. Further targeted studies to identify key pathways and druggable targets will be necessary to prevent or treat PARDS.

## Methods

### Study design and ethics

This study was conducted in a 16-bedded, multidisciplinary PICU at KK Women’s and Children’s Hospital, Singapore from November 2018 to October 2019 (1 year). The study protocol has been approved by the institutional review board (Singhealth Centralised Institutional Review Board reference number: 2017-3076), informed consent was obtained from a parent and/or legal guardian of all subjects before entrance into the study and all methods were carried out in accordance with relevant guidelines and regulations. Reporting was conducted in compliance to the STROBE (strengthening the reporting of observational studies in epidemiology) guidelines^40^.

### Patients

We prospectively studied 16 patients with direct PARDS (8 non-severe and 8 severe) who fulfilled the Pediatric Acute Lung Injury Consensus Conference (PALICC) criteria on two consecutive arterial blood gases four hours apart^3^. In brief, the PALICC criteria include (1) the presence of a known clinical insult within 7 days of PARDS, (2) respiratory failure not fully explained by cardiac failure or fluid overload, (3) new infiltrates on chest imaging consistent with acute pulmonary parenchymal disease, and (4) OI > / = 4. If an arterial blood gas was not available for calculation of OI, the oxygenation saturation index (OSI > / = 5) was used instead. Patients entered the study within 24 h of developing PARDS. There were no patients who had “do-not-resuscitate” orders.

### Definitions and severity scoring

Sepsis and organ dysfunction were defined according to the International Pediatric Sepsis Consensus Conference^41^. Overall, the disease severity on admission was estimated by the Pediatric Index of Mortality 2 (PIM 2)^20^ and Pediatric Logistic Organ Dysfunction (PELOD)^21^ scores, where a higher score indicates greater severity.. Patients were followed up until PICU discharge or 28 days, whichever earlier. Those who survived to PICU discharge or 28 days were classified as survivors. Ventilator free days (VFD) were defined as days alive and free from invasive mechanical ventilation up to 28 days^42^. Intensive care unit free days (IFD) were defined as days alive and discharged from intensive care up to 28 days.

### Blood sampling and processing

The first blood sample (timepoint 1—early PARDS) was obtained within 24 h of developing PARDS^43^. A second sample (timepoint 2—late PARDS) was obtained prior to extubation. If a patient remained intubated for a non-pulmonary indication, the second sample was obtained when the managing physician deemed that the patient was ready for extubation from the pulmonary standpoint. In non-survivors, the second sample were taken prior to death.

Blood was obtained from indwelling arterial cannulas or central venous lines, and collected into EDTA vacutainer tubes. Blood samples were transported to the laboratory within 2 h and centrifuged at 596×*g* for 15 min. The supernatant was collected and centrifuged again at 2095×*g* for 10 min. The plasma was then aspirated and stored at − 80 °C.

### Deep tracheal lavage fluid sampling and processing

Deep tracheal lavage (DTL) fluid was obtained concurrently with the blood samples. Open suction through the endotracheal tube with a sterile appropriately sized suction catheter was performed. Here, the patient was transiently disconnected from the ventilator at the endotracheal tube end, the suction catheter was advanced as far as possible and wedged. 1–3 ml of normal saline was instilled through the catheter and subsequently suctioned at 80–100 cmH_2_O into a sterile trap bottle. Collected fluid was then transported to the laboratory within 2 h and centrifuged at 300×*g* for 10 min. The cell free supernatant was then collected and stored at − 80 °C. For the DTL fluid samples, the urea dilution method was used to standardise lavage fluid concentration^44^.

### Cytokine profiling

The levels of 45 cytokines [eotaxin (CCL11), IL-8, IP-10 (CXCL10), MCP-1 (CCL2), MIP1α (CCL3), MIP-1β (CCL4), SDF-1α (CXCL12), RANTES (CCL5), IFNγ, IL-12p70, IL-13, IL-1β, TNF-α, IL-4, IL-6, GM-CSF, IL-18, IL-10, IL-17A, Il-27, IFN-α, IL-15, IL-1α, IL-1RA, IL-7, brain-derived neurotrophic factor (BDNF), basic nerve growth factor (bNGF), epidermal growth factor (EGF), hepatic growth factor (HGF), leukemia inhibitory factor (LIF), platelet-derived growth factor BB (PDGF-BB), placental growth factor 1 (PIGF-1), stem cell factor (SCF), vascular endothelial growth factor A (VEGF-A), VEGF-D, GRO-alpha (CXCL1), IL-2, IL-5, IL-21, IL-22, IL-23, IL-9, IL-31, TNF-β, fibroblast growth factor (FGF-2)] were measured in the plasma and cell-free DTL fluid using a Procartaplex 45-plex Human Cytokine and Chemokine Panel (Luminex) as described earlier^22^. Briefly, 10 μL of DTL or serum was added to 10 μL of primary antibody-conjugated, magnetic beads on a 96 DropArray plate (Curiox Biosystems, Singapore) and mixed on a plate shaker at 450 rpm for 120 min at 25 °C while being protected from light. Subsequently, the plate was washed three times with wash buffer using the LT210 Washing Station (Curiox) before 5 μL of the secondary antibody was added to each well and incubation done on a plate shaker set at 450 rpm for 30 min at 25 °C with protection of the plate from light. The plate was washed three times with the wash buffer, and 10 μL of streptavidin–phycoerythrin was then added to each well and mixed at 450 rpm for 30 min at 25 °C with protection of the plate from light. The plate was washed with wash buffer three times prior to its transfer to a 96 conical well microtiter plate with 60 μL of reading buffer per well for data acquisition using the Bio-Plex Luminex 200 (BioRad). The beads are classified by the red classification laser (635 nm) into its distinct sets, while a green reporter laser (532 nm) excites the phycoerythrin, a fluorescent reporter tag bound to the detection antibody. Quantitation of the 45 cytokines in each sample was then determined by extrapolation to a six-or seven-point standard curve using five-parameter logistic regression modelling^45^. Calibrations and validations were performed prior to runs and on a monthly basis respectively. DTL and serum cytokine levels are captured as pg/mL for both timepoints of PARDS. All samples were run in duplicates and the average was reported. Cytokines with more than 50% missing data were not analysed. The cytokine concentrations had an intra-assay coefficient of variation within 15%.

10 out of 45 cytokines which had greater than 50% of data points out of dynamic range were not analysed (FGF-2, GRO-alpha, IL-2, IL-21, IL-2, IL-23, IL-31, IL-5, IL-9, TNF-β). Concentrations of all analysed cytokines were higher in DTL compared to plasma, except IFN-γ, IL-18 and PDGFBB (Supplementary Table 6).

### Statistical analysis

Clinical characteristics of patients in the severe and non-severe PARDS groups were first compared using the Fisher-exact and Student’s t-test for categorical and continuous variables, respectively. These were reported as counts (percentages) and mean (standard deviation). Subsequently, to find out which of the cytokines were most related to disease severity, we compared DTL cytokine concentration in the following groups; (i) severe and non-severe PARDS, and (ii) between longitudinal samples taken during the course of PARDS. All data were Log2 transformed prior to analysis and normality was assumed. Pearson’s correlation coefficient was used to quantify the relationship between DTL cytokine concentration and pulmonary severity of illness (OI), and repeated for systemic severity of illness (PIM2 and PELOD). Statistically significant comparison of cytokines and correlation were graphically represented by box and whisker plots and scatterplots with an interposed linear regression line, respectively.

Cytokine signatures were further analysed by partial least squares regression (PLSR) modelling (Unscrambler X version 10.1) PLSR is a form of supervised regression method performed to visualize the global cytokine changes in PARDS. Full cross-validation was applied in PLSR to increase model performance and for the calculation of coefficient regression values. Regression coefficient plots were then created to identify the top ten cytokines that are associated with severity and the early timepoint. Statistical analysis and graphical representation were performed using GraphPad Prism 8 (GraphPad Software, USA) and STATA version 15.0 statistical software (StataCorp, College Station, Texas). All *t*-tests were two-tailed and statistical significance was taken as *p* < 0.05.

### Pathway enrichment analysis

PLSR coefficient-derived cytokines were then imputed into the Database for Annotation, Visualization and Integrated Discovery database (DAVID Bioinformatics Resources 6.8), and cross-referenced against Gene Ontology (GO) and Gene Association Disease (GAD) databases for biological process enrichment analysis as previously described^22^. *p* < 0.05 based on Fisher Exact analysis and fold change > 10 were imposed as enriched pathways.

## Supplementary Information

Supplementary Information.

## References

[1] Schouten LR (2016). Incidence and mortality of acute respiratory distress syndrome in children: A systematic review and meta-analysis. Crit. Care Med..

[2] Wong JJ (2017). Risk stratification in pediatric acute respiratory distress syndrome: A multicenter observational study. Crit. Care Med..

[3] Pediatric Acute Lung Injury Consensus Conference Group (2015). Pediatric acute respiratory distress syndrome: Consensus recommendations from the pediatric acute lung injury consensus conference. Pediatr. Crit. Care Med..

[4] Wong JJ (2020). Characteristics and trajectory of patients with pediatric acute respiratory distress syndrome. Pediatr. Pulmonol..

[5] Kneyber MC, Brouwers AG, Caris JA, Chedamni S, Plotz FB (2008). Acute respiratory distress syndrome: Is it underrecognized in the pediatric intensive care unit?. Intensive Care Med..

[6] Ashbaugh DG, Bigelow DB, Petty TL, Levine BE (1967). Acute respiratory distress in adults. Lancet.

[7] Matthay MA (2019). Acute respiratory distress syndrome. Nat. Rev. Dis. Primers..

[8] Wong JJM, Leong JY, Lee JH, Albani S, Yeo JG (2019). Insights into the immuno-pathogenesis of acute respiratory distress syndrome. Ann. Transl. Med..

[9] Thille AW (2013). Chronology of histological lesions in acute respiratory distress syndrome with diffuse alveolar damage: A prospective cohort study of clinical autopsies. Lancet Respir. Med..

[10] Park WY (2001). Cytokine balance in the lungs of patients with acute respiratory distress syndrome. Am. J. Respir. Crit. Care Med..

[11] Meduri GU (1995). Persistent elevation of inflammatory cytokines predicts a poor outcome in ARDS. Plasma IL-1 beta and IL-6 levels are consistent and efficient predictors of outcome over time. Chest.

[12] Schutte H (1996). Bronchoalveolar and systemic cytokine profiles in patients with ARDS, severe pneumonia and cardiogenic pulmonary oedema. Eur. Respir. J..

[13] Ranieri VM (2012). Acute respiratory distress syndrome: The Berlin Definition. JAMA.

[14] Bellani G (2016). Epidemiology, patterns of care, and mortality for patients with acute respiratory distress syndrome in intensive care units in 50 countries. JAMA.

[15] Kneyber MC, Zhang H, Slutsky AS (2014). Ventilator-induced lung injury. Similarity and differences between children and adults. Am. J. Respir. Crit. Care Med..

[16] Dobyns EL, Eells PL, Griebel JL, Abman SH (1999). Elevated plasma endothelin-1 and cytokine levels in children with severe acute respiratory distress syndrome. J. Pediatr..

[17] Zinter MS (2017). Incorporating inflammation into mortality risk in pediatric acute respiratory distress syndrome. Crit. Care Med..

[18] McKeone DJ (2020). Cytokine panels and pediatric acute respiratory distress syndrome: A translational investigation*. Pediatr. Crit. Care Med..

[19] Connors TJ (2016). Airway CD8(+) T cells are associated with lung injury during infant viral respiratory tract infection. Am. J. Respir. Cell Mol. Biol..

[20] Slater A, Shann F, Pearson G (2003). PIM2: A revised version of the Paediatric Index of Mortality. Intensive Care Med..

[21] Leteurtre S (2003). Validation of the paediatric logistic organ dysfunction (PELOD) score: Prospective, observational, multicentre study. Lancet.

[22] Zhou J (2020). Peritoneal fluid cytokines reveal new insights of endometriosis subphenotypes. Int. J. Mol. Sci..

[23] Vignali DAA, Kuchroo VK (2012). IL-12 family cytokines: Immunological playmakers. Nat. Immunol..

[24] Gee K, Guzzo C, Che Mat NF, Ma W, Kumar A (2009). The IL-12 family of cytokines in infection, inflammation and autoimmune disorders. Inflamm. Allergy Drug Targets.

[25] Iwasaki Y, Fujio K, Okamura T, Yamamoto K (2015). Interleukin-27 in T cell immunity. Int. J. Mol. Sci..

[26] Kalliolias GD, Ivashkiv LB (2008). IL-27 activates human monocytes via STAT1 and suppresses IL-10 production but the inflammatory functions of IL-27 are abrogated by TLRs and p38. J. Immunol..

[27] Muir R (2016). Innate lymphoid cells are the predominant source of IL-17A during the early pathogenesis of acute respiratory distress syndrome. Am. J. Respir. Crit. Care Med..

[28] Nembrini C, Marsland BJ, Kopf M (2009). IL-17-producing T cells in lung immunity and inflammation. J. Allergy Clin. Immunol..

[29] Gu C, Wu L, Li X (2013). IL-17 family: Cytokines, receptors and signaling. Cytokine.

[30] Ye P (2001). Requirement of interleukin 17 receptor signaling for lung CXC chemokine and granulocyte colony-stimulating factor expression, neutrophil recruitment, and host defense. J. Exp. Med..

[31] Kawaguchi M (2007). The IL-17F signaling pathway is involved in the induction of IFN-γ–inducible protein 10 in bronchial epithelial cells. J. Allergy Clin. Immunol..

[32] Balamayooran G, Batra S, Balamayooran T, Cai S, Jeyaseelan S (2011). Monocyte chemoattractant protein 1 regulates pulmonary host defense via neutrophil recruitment during *Escherichia coli* infection. Infect. Immun..

[33] Lang S (2017). CXCL10/IP-10 neutralization can ameliorate lipopolysaccharide-induced acute respiratory distress syndrome in rats. PLoS ONE.

[34] Laan M (2003). A role of GM-CSF in the accumulation of neutrophils in the airways caused by IL-17 and TNF-alpha. Eur. Respir. J..

[35] Traber KE (2017). Myeloid-epithelial cross talk coordinates synthesis of the tissue-protective cytokine leukemia inhibitory factor during pneumonia. Am. J. Physiol. Lung Cell. Mol. Physiol..

[36] Knight DA (1999). Leukemia inhibitory factor (LIF) and LIF receptor in human lung. Am. J. Respir. Cell Mol. Biol..

[37] Ikezono T (1997). Leukemia inhibitory factor induces the 85-kDa cytosolic phospholipase A2 gene expression in cultured human bronchial epithelial cells. Biochem. Biophys. Acta..

[38] Quinton LJ (2012). Leukemia inhibitory factor signaling is required for lung protection during pneumonia. J. Immunol..

[39] Jorens PG (1996). High levels of leukaemia inhibitory factor in ARDS. Cytokine.

[40] von Elm E (2007). The Strengthening the Reporting of Observational Studies in Epidemiology (STROBE) statement: Guidelines for reporting observational studies. Prev. Med..

[41] Goldstein B, Giroir B, Randolph A (2005). International pediatric sepsis consensus conference: Definitions for sepsis and organ dysfunction in pediatrics. Pediatr. Crit. Care Med..

[42] Yehya N, Harhay MO, Curley MAQ, Schoenfeld DA, Reeder RW (2019). Reappraisal of ventilator-free days in critical care research. Am. J. Respir. Crit. Care Med..

[43] Yehya N, Thomas NJ, Khemani RG (2018). Risk stratification using oxygenation in the first 24 hours of pediatric acute respiratory distress syndrome. Crit. Care Med..

[44] de Blic J (2000). Bronchoalveolar lavage in children. ERS Task Force on bronchoalveolar lavage in children. European Respiratory Society. Eur. Respir. J..

[45] Gottschalk PG, Dunn JR (2005). The five-parameter logistic: A characterization and comparison with the four-parameter logistic. Anal. Biochem..

