# Supplementary material for: Large scale cytokine profiling uncovers elevated IL12-p70 and IL-17A in severe pediatric acute respiratory distress syndrome
Source: Sci Rep. 2021 Jul 8;11:14158. doi: 10.1038/s41598-021-93705-8 (PMC8266860; doi:10.1038/s41598-021-93705-8)
Supplement: Supplementary file 1 — Supplementary Information. [file 41598_2021_93705_MOESM1_ESM.docx]

**Supplementary Digital Content**

| **Supplementary Tables and Figures** | **Page** |
| --- | --- |
| Table 1A: Respiratory cytokine concentration in PARDS comparing timepoint 1 and 2 | 2 |
| Table 1B: Plasma cytokine concentration in PARDS comparing timepoint 1 and 2 | 3 |
| Table 2: Top 10 pathways enriched in patients with pediatric acute respiratory distress syndrome from DAVID functional annotation module analysis | 4 |
| Table 3A: Respiratory cytokine concentration in severe and non-severe PARDS at timepoint 1 | 6 |
| Table 3B: Plasma cytokine concentration in severe and non-severe PARDS at timepoint 1 | 7 |
| Table 4: Correlation matrix of significantly correlated respiratory and plasma cytokines with severity scores | 8 |
| Table 5A: Respiratory cytokine concentration in severe and non-severe PARDS at timepoint 2 | 9 |
| Table 5B: Plasma cytokine concentration in severe and non-severe PARDS at timepoint 2 | 10 |
| Table 6: Overall cytokine concentrations in deep tracheal lavage and plasma in PARDS | 11 |
| Figure 1: Correlation between acute phase plasma cytokines and the oxygenation index | 12 |
|  |  |

**Supplementary Table 1A: Respiratory cytokine concentration in PARDS comparing timepoint 1 and 2**

| Cytokine concentration, pg/ml (log2) | Non-severe PARDS | | | Severe PARDS | | |
| --- | --- | --- | --- | --- | --- | --- |
|  | Time point 1 | Time point 2 | T test | Time point 1 | Time point 2 | T test |
| BDNF | 6.5 (1.1) | 6.8 (1.5) | 0.8042 | 6.7 (2.2) | 7.5 (2.1) | 0.5024 |
| BNGF | 10.4 (1.6) | 10.2 (1.6) | 0.7933 | 10.7 (2.2) | 11.6 (2.1) | 0.4564 |
| EGF | 12.6 (2.1) | 14.3 (2.7) | 0.1856 | 14.4 (2.1) | 13.0 (2.3) | 0.2608 |
| Eotaxin | 9.6 (2.6) | 11.0 (2.1) | 0.2645 | 11.1 (2.1) | 11.0 (2.5) | 0.9587 |
| GM-CSF | 6.9 (2.8) | 4.6 (1.5) | 0.1094 | 8.4 (1.9) | 8.8 (3.3) | 0.7855 |
| HGF | 13.9 (1.9) | 13.7 (2.0) | 0.7872 | 15.6 (3.4) | 14.8 (3.6) | 0.6751 |
| IFN-alpha | 3.7 (3.1) | 1.6 (1.0) | 0.2354 | 4.1 (2.3)) | 4.8 (1.7) | 0.5788 |
| IFN-gamma | 8.5 (2.3) | 7.8 (1.5) | 0.5006 | 9.7 (5.3) | 9.4 (5.7) | 0.9085 |
| IL-10 | 7.3 (1.7) | 5.0 (2.6) | 0.0934 | 8.7 (2.1) | 9.1 (6.1) | 0.8725 |
| IL-13 | 3.5 (1.9) | 3.2 (2.2) | 0.8302 | 4.8 (3.8) | 4.5 (3.3) | 0.8912 |
| IL-15 | 6.8 (0.8) | 5.7 (1.5) | 0.1187 | 8.1 (4.7) | 7.6 (2.2) | 0.8310 |
| IL-17A | 3.6 (3.0) | 2.9 (0.4) | 0.7682 | 8.6 (2.1) | 7.1 (1.7) | 0.2763 |
| IL-18 | 8.4 (1.9) | 8.0 (1.6) | 0.6141 | 8.8 (3.5) | 9.2 (4.3) | 0.8530 |
| IL-1alpha | 6.6 (1.7) | 6.5 (2.0) | 0.8929 | 6.6 (2.8) | 7.8 (2.3) | 0.3972 |
| IL-1beta | 6.7 (3.2) | 9.7 (3.2) | 0.0808 | 9.0 (5.0) | 9.5 (4.4) | 0.8275 |
| IL-1RA | 18.3 (2.1) | 18.7 (1.6) | 0.6476 | 18.5 (3.1) | 18.3 (3.1) | 0.9333 |
| IL-27 | 11.0 (2.7) | 9.8 (2.1) | 0.3970 | 11.0 (2.8) | 12.6 (2.0) | 0.2904 |
| IL-4 | 3.8 (2.9) | 5.8 (0.5) | 0.3962 | 7.0 (2.7) | 8.3 (2.9) | 0.4981 |
| IL-6 | 12.6 (2.0) | 13.0 (2.4) | 0.7294 | 14.8 (3.7) | 13.3 (3.2) | 0.4659 |
| IL-7 | 6.6 (1.3) | 6.9 (1.7) | 0.7719 | 7.1 (1.4) | 6.5 (1.2) | 0.3907 |
| IL-8 | 13.8 (4.2) | 13.5 (3.5) | 0.8729 | 15.6 (4.5) | 15.6 (3.0) | 0.9901 |
| IL-12p70 | 4.2 (1.3) | 3.7 (1.7) | 0.5658 | 7.9 (3.2) | 7.3 (3.3) | 0.7875 |
| IP-10 | 13.6 (1.9) | 13.8 (2.1) | 0.8343 | 14.3 (1.5) | 15.3 (1.1) | 0.1893 |
| LIF | 10.1 (1.4) | 9.3 (1.8) | 0.3522 | 11.4 (3.6) | 10.7 (4.3) | 0.7512 |
| MCP-1 | 11.8 (3.6) | 13.6 (2.6) | 0.2787 | 14.3 (3.4) | 14.6 (2.8) | 0.8917 |
| MIP-1alpha | 9.0 (1.5) | 10.1 (1.9) | 0.1875 | 10.6 (3.8) | 11.1 (3.7) | 0.7992 |
| MIP-1beta | 12.4 (1.9) | 13.5 (2.1) | 0.2871 | 14.6 (3.7) | 14.2 (3.1) | 0.8406 |
| PDGFBB | 9.6 (1.5) | 8.7 (1.8) | 0.3668 | 11.2 (2.2) | 7.7 (3.8) | 0.0736 |
| PIGF-1 | 7.3 (1.1) | 7.1 (2.1) | 0.7601 | 7.9 (2.2) | 8.7 (1.8) | 0.4978 |
| RANTES | 9.3 (1.7) | 9.1 (1.7) | 0.7930 | 9.6 (2.5) | 10.2 (2.5) | 0.6969 |
| SCF | 6.5 (1.6) | 6.5 (1.8) | 0.9418 | 8.3 (2.8) | 7.7 (2.3) | 0.6457 |
| SDF-1a | 14.0 (1.8) | 13.8 (2.1) | 0,8450 | 14.8 (2.4) | 15.8 (2.1) | 0.4400 |
| TNF-alpha | 7.9 (2.9) | 8.1 (2.3) | 0.8990 | 9.6 (4.1) | 13.9 (5.5) | 0.2309 |
| VEGF-A | 16.0 (1.7) | 16.9 (1.7) | 0.3386 | 15.6 (2.2) | 17.2 (1.7) | 0.1439 |
| VEGF-D | 6.5 (0.6 ) | 6.3 (1.7) | 0.8260 | 7.7 (4.0) | 9.9 (1.9) | 0.3404 |

PARDS – pediatric acute respiratory distress syndrome

**Supplementary Table 1B: Plasma cytokine concentration in PARDS comparing timepoint 1 and 2**

| Cytokine concentration, pg/ml (log2) | Non-severe PARDS | | | Severe PARDS | | |
| --- | --- | --- | --- | --- | --- | --- |
|  | Time point 1 | Time point 2 | T test | Time point 1 | Time point 2 | T test |
| BDNF | 6.4 (2.1) | 7.2 (1.3) | 0.3620 | 5.2 (1.2) | 4.5 (1.9) | 0.4673 |
| BNGF | 8.2 (1.5) | 8.4 (1.2) | 0.8603 | 7.7 (1.5) | 7.2 (2.1) | 0.6378 |
| EGF | 3.7 (3.6) | 3.2 (1.3) | 0.7488 | 3.5 (1.3) | 3.2 (1.0) | 0.5830 |
| Eotaxin | 6.4 (1.3) | 6.6 (1.0) | 0.6435 | 6.6 (1.7) | 7.1 (1.8) | 0.5562 |
| GM-CSF | 3.8 (2.5) | 5.5 91.8) | 0.2193 | 5.2 (2.7) | 5.8 (1.6) | 0.6012 |
| HGF | 9.6 (1.3) | 9.2 (0.9) | 0.5954 | 11.2 (0.9) | 10.3 (1.0) | 0.1346 |
| IFN-alpha | 0.4 (3.0) | 2.9 (1.3) | 0.1608 | 2.8 (1.5) | 2.1 (1.4) | 0.4621 |
| IFN-gamma | 8.1 (1.1) | 7.8 (1.0) | 0.4601 | 9.0 (1.8) | 8.1 (2.1) | 0.3807 |
| IL-10 | 2.5 (3.2) | 3.5 (0.6) | 0.4669 | 3.8 92.1) | 3.2 94.6) | 0.7441 |
| IL-13 | 2.1 (1.0) | 2.1 (1.0) | 0.9274 | 2.8 (1.2) | 2.5 (1.6) | 0.6888 |
| IL-15 | 5.4 (1.9) | 5.1 (2.3) | 0.8178 | 5.2 (2.5) | 5.5 (1.7) | 0.7627 |
| IL-17A | 2.8 (3.5) | 4.2 (1.5) | 0.4376 | 4.7 (1.6) | 4.3 (1.8) | 0.6400 |
| IL-18 | 8.0 (1.1) | 7.5 (1.0) | 0.3573 | 8.1 (1.6) | 8.0 (2.3) | 0.9203 |
| IL-1alpha | 0.7 (1.8) | 0.0 (1.9) | 0.5318 | 0.7 (1.1) | 0.9 (2.7) | 0.9142 |
| IL-1beta | 1.9 (1.7) | 1.5 (1.3) | 0.6416 | 1.6 (1.8) | 1.2 (2.3) | 0.6947 |
| IL-1RA | 11.2 (3.1) | 9.5 (2.9) | 0.3101 | 12.5 (2.5) | 11.1 (3.3) | 0.3808 |
| IL-27 | 8.7 (2.6) | 9.5 (2.4) | 0.6384 | 9.6 (2.5) | 10.0 (1.6) | 0.7125 |
| IL-4 | 3.4 (1.4) | 4.0 (2.2) | 0.6166 | 5.0 (2.2) | 5.9 (1.5) | 0.4340 |
| IL-6 | 6.2 (2.8) | 6.2 (2.6) | 0.9873 | 7.3 (3.1) | 5.5 (2.1) | 0.2682 |
| IL-7 | 2.5 (0.7) | 1.6 91.9) | 0.2991 | 2.0 (1.0) | 1.7 (1.7) | 0.7889 |
| **IL-8** | **8.8 (0.0)** | **5.2 (1.3)** | **0.0185** | 5.0 (3.2) | 3.5 (2.3) | 0.3475 |
| IL-12p70 | 3.2 (1.0) | 2.9 (1.1) | 0.7076 | 3.9 (1.5) | 3.4 (2.3) | 0.6163 |
| IP-10 | 10.7 (1.8) | 9.8 (1.7) | 0.3657 | 11.0 (3.0) | 10.3 (3.0) | 0.6790 |
| LIF | 3.6 (2.7) | 4.1 (2.3) | 0.7149 | 5.7 (1.4) | 5.3 (1.4) | 0.5941 |
| MCP-1 | 7.1 (0.9) | 7.4 (1.0) | 0.5426 | 6.6 (1.0) | 7.3 (1.0) | 0.2754 |
| MIP-1alpha | 4.9 (1.2) | 4.8 (0.7) | 0.9008 | 5.1 (0.9) | 5.1 (1.6) | 0.9197 |
| MIP-1beta | 7.9 (1.4) | 7.7 (0.5) | 0.7542 | 7.8 (0.8) | 7.7 (1.4) | 0.8338 |
| PDGFBB | 8.6 (1.3) | 9.0 (0.6) | 0.5204 | 8.8 (1.3) | 8.9 (1.4) | 0.8520 |
| PIGF-1 | 4.0 (0.8) | 3.4 (1.5) | 0.4054 | 2.9 (0.7) | 2.4 (1.3) | 0.4192 |
| RANTES | 7.5 (2.1) | 7.6 (0.7) | 0.9686 | 7.6 (0.9) | 6.3 (1.6) | 0.1893 |
| SCF | 4.4 (1.1) | 4.4 (1.3) | 0.9545 | 5.3 (1.4) | 5.6 (1.4) | 0.7132 |
| SDF-1a | 10.2 (0.5) | 10.3 (0.4) | 0.7635 | 10.2 (0.9) | 9.9 (0.3) | 0.3999 |
| TNF-alpha | 3.3 (1.9) | 3.3 (1.9) | 0.9648 | 4.9 (1.8) | 5.2 (2.1) | 0.8100 |
| VEGF-A | 9.9 (1.0) | 9.6 (0.7) | 0.4905 | 10.0 (2.1) | 10.1 (2.5) | 0.9429 |
| VEGF-D | 3.9 (2.4) | 5.4 (2.5) | 0.2986 | 6.1 (1.4) | 6.2 (2.2) | 0.9343 |

PARDS – pediatric acute respiratory distress syndrome

**Supplementary Table 2: Top 10 biological pathways enriched in patients with pediatric acute respiratory distress syndrome from Database for Annotation, Visualization and Integrated Discovery (DAVID) database functional annotation module analysis**

| Term/gene function | Genes | Fold Enrichment | P value | FDR |
| --- | --- | --- | --- | --- |
| GOTERM_BP_DIRECT | | | | |
| GO:0006955  Immune response | MIP-1β/ CCL4 , IL-6,  IL-4, IL-1β, MCP-1/ CCL2 , IL-12p70/ IL-12A  IL-17A | 27.92019 | 1.89 E-8 | 6.82 E-6 |
| GO:0042102  Positive regulation of T cell proliferation | IL-6, IL-4, IL-1β, IL-12p70/ IL-12A | 111.94666 | 3.59 E-6 | 6.48 E-4 |
| GO:0071347  Cellular response to interleukin-1 | MIP-1β/ CCL4, IL-6  MCP-1/ CCL2, IL-17A | 94.60281 | 5.97 E-6 | 7.19 E-4 |
| GO:0006954  Inflammatory response | MIP-1β/ CCL4, IL-6, IL-1β, MCP-1/ CCL2, IL-17A | 22.15303 | 2.94 E-5 | 0.00266 |
| GO:0042832  Defence response to protozoan | IL-6, IL-4, IL-12p70/ IL-12A | 265.13684 | 4.35 E-5 | 0.00314 |
| GO:0097192  Extrinsic apoptotic signalling pathway in absence of ligand | SCF/ KITLG, IL-4,  IL-1 β | 148.164706 | 1.42 E-4 | 0.008543 |
| GO:0002548  Monocyte chemotaxis | MIP-1β/ CCL4, IL-6,  MCP-1/ CCL2 | 119.94286 | 2.17 E-4 | 0.01121 |
| GO:0000165  MAPK cascade | EGF, SCF/ KITLG,  IL-1β, MCP-1/ CCL2 | 25.63664 | 2.94 E-4 | 0.01328 |
| GO:0030593  Neutrophil chemotaxis | MIP-1β/ CCL4 (6351)  IL-1β (3553)  MCP-1/ CCL2 (6347) | 76.32727 | 5.38 E-4 | 0.02123 |
| GO:0035690  Cellular response to drug | MIP-1β/ CCL4,, IL-1β, MCP-1/ CCL2 | 73.00870 | 5.88 E-4 | 0.02123 |
| GAD_DISEASE | | | | |
| Asthma\| Bronchiolitis, Viral\| Respiratory Syncytial Virus Infections | MIP-1β/ CCL4, IL-6,  IL-4, IL-1β, MCP-1/ CCL2, IL-12p70/ IL-12A,  IL-17A | 44.50833 | 1.13 E-9 | 4.11 E-7 |
| Respiratory syncytial virus bronchiolitis | MIP-1β/ CCL4, IL-6,  IL-4, IL-1β, MCP-1/ CCL2, IL-12p70/ IL-12A,  IL-17A | 44.50833 | 1.14 E-9 | 4.11 E-7 |
| Bronchiolitis, Viral\| Respiratory Syncytial Virus Infections | MIP-1β/ CCL4, IL-6,  IL-4, IL-1β, MCP-1/ CCL2, IL-12p70/ IL-12A IL-17A | 43.03175 | 1.39 E-9 | 4.11 E-7 |
| Juvenile arthritis | IL-6, IL-4, IL-1β,  MCP-1/ CCL2,  IL-12p70/ IL-12A | 223.63793 | 2.52 E-9 | 5.58 E-7 |
| Asthma | IL-4, IL-1β, MCP-1/ CCL2, IL-12p70/ IL-12A, IL-17A | 65.95423 | 7.00 E-9 | 1.24 E-6 |
| Helicobacter Infections\| Stomach Neoplasms | IL-6, IL-4, IL-1β,  IL-12p70/ IL-12A,  IL-17A | 150.825581 | 1.30 E-8 | 1.92 E-6 |
| Atherosclerosis\| Inflammation\| Retinal Vein Occlusion | IL-6, IL-4, IL-1β,  MCP-1/ CCL2 | 518.84000 | 2.77 E-8 | 3.50 E-6 |
| Tuberculosis, Pulmonary | IL-6, IL-4, IL-1β,  MCP-1/ CCL2,  IL-12p70/ IL-12A | 113.78070 | 4.15 E-8 | 4.60 E-6 |
| Arthritis | IL-6, IL-4, IL-1β, MCP-1/ CCL2, IL-12p70/ IL-12A | 109.92373 | 4.782 E-8 | 4.71 E-6 |
| Desensitization in solid organ transplant recipients | IL-6, IL-4, IL-1β,  IL-12p70/ IL-12A | 399.10769 | 6.58 E-8 | 5.30 E-6 |

FDR – false discovery rate, GO – gene ontology database, GAD – genetic association disease database

**Supplementary Table 3A: Respiratory cytokine concentration in severe and non-severe PARDS at timepoint 1**

| Cytokine concentration, pg/ml (log2) | Non-severe PARDS (n=8) | Severe PARDS (n=8) | T Test |
| --- | --- | --- | --- |
|  | Mean (SD) | Mean (SD) |  |
| BDNF | 6.5 (1.1) | 6.7 (2.2) | 0.8307 |
| BNGF | 10.4 (1.6) | 10.7 (2.2) | 0.7133 |
| EGF | 12.6 (2.1) | 14.4 (2.1) | 0.1313 |
| Eotaxin | 9.6 (2.6) | 11.1 (2.1) | 0.2543 |
| GM-CSF | 6.9 (2.8) | 8.4 (1.9) | 0.3632 |
| HGF | 13.9 (1.9) | 15.6 (3.4) | 0.2500 |
| IFN-alpha | 3.7 (3.1) | 4.1 (2.3) | 0.7729 |
| IFN-gamma | 8.5 (2.3) | 9.7 (5.3) | 0.5507 |
| IL-10 | 7.3 (1.7) | 8.7 (2.1) | 0.2222 |
| IL-13 | 3.5 (1.9) | 4.8 (3.8) | 0.4046 |
| IL-15 | 6.8 (0.8) | 8.1 (4.7) | 0.5415 |
| **IL-17A** | **3.6 (3.0)** | **8.6 (2.1)** | **0.0455** |
| IL-18 | 8.4 (1.9) | 8.8 (3.5) | 0.8110 |
| IL-1alpha | 6.6 (1.7) | 6.6 (2.8) | 0.9955 |
| IL-1beta | 6.7 (3.2) | 9.0 (5.0) | 0.3128 |
| IL-1RA | 18.3 (2.1) | 18.5 (3.1) | 0.8806 |
| IL-27 | 11.0 (2.7) | 11.0 (2.8) | 0.9714 |
| IL-4 | 3.8 (2.9) | 7.0 (2.7) | 0.0919 |
| IL-6 | 12.6 (2.0) | 14.8 (3.7) | 0.1739 |
| IL-7 | 6.6 (1.3) | 7.1 (1.4) | 0.4993 |
| IL-8 | 13.8 (4.2) | 15.6 (4.5) | 0.4196 |
| **IL-12p70** | **4.2 (1.3)** | **7.9 (3.2)** | **0.0188** |
| IP-10 | 13.6 (1.9) | 14.3 (1.5) | 0.4151 |
| LIF | 10.1 (1.4) | 11.4 (3.6) | 0.3633 |
| MCP-1 | 11.8 (3.6) | 14.3 (3.4) | 0.1856 |
| MIP-1alpha | 9.0 (1.5) | 10.6 (3.8) | 0.2840 |
| MIP-1beta | 12.4 (1.9) | 14.6 (3.7) | 0.1614 |
| PDGFBB | 9.6 (1.5) | 11.2 (2.2) | 0.1386 |
| PIGF-1 | 7.3 (1.1) | 7.9 (2.2) | 0.5129 |
| RANTES | 9.3 (1.7) | 9.6 (2.5) | 0.7607 |
| SCF | 6.5 (1.7) | 8.3 (2.8) | 0.1332 |
| SDF-1a | 14.0 (1.8) | 14.8 (2.4) | 0.4956 |
| TNF-alpha | 7.9 (2.9) | 9.6 (4.1) | 0.4882 |
| VEGF-A | 16.0 (1.7) | 15.6 (2.2) | 0.6807 |
| VEGF-D | 6.5 (0.6) | 7.7 (4.0) | 0.5816 |

PARDS – pediatric acute respiratory distress syndrome, SD – standard deviation

**Supplementary Table 3B: Plasma cytokine concentration in severe and non-severe PARDS at timepoint 1**

| Cytokine concentration, pg/ml (log2) | Non-severe PARDS (n=8) | Severe PARDS (n=8) | T Test |
| --- | --- | --- | --- |
|  | Mean (SD) | Mean (SD) |  |
| BDNF | 6.4 (2.1) | 5.2 (1.2) | 0.1819 |
| BNGF | 8.2 (1.5) | 7.7 (1.5) | 0.6149 |
| EGF | 3.7 (3.6) | 3.5 (1.3) | 0.8779 |
| Eotaxin | 6.4 (1.3) | 6.6 (1.7) | 0.7992 |
| GM-CSF | 3.8 (2.5) | 5.2 (2.7) | 0.3354 |
| **HGF** | **9.6 (1.3)** | **11.2 (0.9)** | **0.0257** |
| IFN-alpha | 0.4 (3.0) | 2.8 (1.5) | 0.1151 |
| IFN-gamma | 8.2 (1.1) | 9.0 (1.8) | 0.3123 |
| IL-10 | 2.5 (3.2) | 3.8 (2.1) | 0.4031 |
| IL-13 | 2.1 (1.0) | 2.8 (1.2) | 0.2976 |
| IL-15 | 5.4 (1.9) | 5.2 (2.5) | 0.8484 |
| IL-17A | 2.8 (3.5) | 4.7 (1.6) | 0.2637 |
| IL-18 | 8.0 (1.1) | 8.1 (1.6) | 0.9321 |
| IL-1alpha | 0.7 (1.8) | 0.7 (1.1) | 0.9595 |
| IL-1beta | 1.9 (1.7) | 1.6 (1.8) | 0.7558 |
| IL-1RA | 11.2 (3.1) | 12.5 (2.5) | 0.3713 |
| IL-27 | 8.7 (2.6) | 9.6 (2.5) | 0.5911 |
| IL-4 | 3.4 (2.4) | 5.0 (2.2) | 0.1994 |
| IL-6 | 6.2 (2.8) | 7.3 (3.1) | 0.5106 |
| IL-7 | 2.5 (0.7) | 2.0 (1.0) | 0.2828 |
| IL-8 | 8.8 (0.0) | 5.0 (3.2) | 0.1506 |
| IL-12p70 | 3.2 (1.0) | 3.9 (1.5) | 0.2815 |
| IP-10 | 10.7 (1.8) | 11.0 (3.0) | 0.8228 |
| LIF | 3.6 (2.7) | 5.7 (1.4) | 0.0859 |
| MCP-1 | 7.1 (0.9) | 6.6 (1.0) | 0.3576 |
| MIP-1alpha | 4.9 (1.2) | 5.1 (0.9) | 0.6411 |
| MIP-1beta | 7.9 (1.4) | 7.8 (0.8) | 0.8641 |
| PDGFBB | 8.6 (1.3) | 8.8 (1.3) | 0.7932 |
| **PIGF-1** | **4.0 (0.8)** | **2.9 (0.7)** | **0.0220** |
| RANTES | 7.5 (2.1) | 7.6 (0.9) | 0.9447 |
| SCF | 4.4 (1.1) | 5.3 (1.4) | 0.1941 |
| SDF-1a | 10.2 (0.5) | 10.2 (0.9) | 0.9235 |
| TNF-alpha | 3.3 (1.9) | 4.9 (1.8) | 0.1230 |
| VEGF-A | 9.9 (1.0) | 10.0 (2.1) | 0.9138 |
| VEGF-D | 3.9 (2.4) | 6.1 (1.4) | 0.1186 |

PARDS – pediatric acute respiratory distress syndrome, SD – standard deviation

**Supplementary Table 4: Correlation matrix of significantly correlated respiratory and plasma cytokines with severity scores**

|  | **OI** | | | **PIM 2 score** | | | **PELOD score** | | |
| --- | --- | --- | --- | --- | --- | --- | --- | --- | --- |
| **Early PARDS** | **Cytokines** | **Pearson’s correlation** | **P value** | **Cytokines** | **Pearson’s correlation** | **P value** | **Cytokines** | **Pearson’s correlation** | **P value** |
| **DTL** | IL-1RA | 0.54 | 0.0387 |  |  |  |  |  |  |
|  | IL-6 | 0.53 | 0.0429 |  |  |  |  |  |  |
|  | IP-10 | 0.67 | 0.0067 |  |  |  |  |  |  |
|  | LIF | 0.52 | 0.0452 |  |  |  |  |  |  |
|  | MCP-1 | 0.66 | 0.0074 |  |  |  |  |  |  |
|  | SDF-1A | 0.52 | 0.0493 |  |  |  |  |  |  |
| **Plasma** | IL-6 | 0.66 | 0.0096 | MCP-1 | -0.58 | 0.0370 | IL-27 | -0.60 | 0.0380 |
|  | IL-10 | 0.58 | 0.0357 | SCF | 0.54 | 0.0387 | IL-15 | -0.63 | 0.0211 |
|  | VEGF-A | 0.60 | 0.0170 |  |  |  | HGF | 0.77 | 0.0019 |
| **Late PARDS** | **Cytokines** | **Pearson’s correlation** | **P value** | **Cytokines** | **Pearson’s correlation** | **P value** | **Cytokines** | **Pearson’s correlation** | **P value** |
| **DTL** |  |  |  |  |  |  |  |  |  |
| **Plasma** |  |  |  |  |  |  | HGF | 0.76 | 0.0070 |

OI – oxygenation index, PARDS – pediatric acute respiratory distress syndrome, PIM 2 score – Pediatric Index of Mortality 2 score, PELOD – pediatric logistic organ dysfunction score

**Supplementary Table 5A: Respiratory cytokine concentration in severe and non-severe PARDS at timepoint 2**

| Cytokine concentration, pg/ml (log2) | Non-severe PARDS (n=8) | Severe PARDS (n=8) | T Test |
| --- | --- | --- | --- |
|  | Mean (SD) | Mean (SD) |  |
| BDNF | 6.7 (1.5) | 7.5 (2.1) | 0.3905 |
| BNGF | 10.2 (1.6) | 11.6 (2.1) | 0.1501 |
| EGF | 14.3 (2.7) | 13.0 (2.3) | 0.3335 |
| Eotaxin | 11.0 (2.1) | 11.0 (2.5) | 0.9741 |
| **GM-CSF** | **4.6 (1.5)** | **8.8 (3.3)** | **0.0182** |
| HGF | 13.7 (2.0) | 14.8 (3.6) | 0.4507 |
| **IFN-alpha** | **1.6 (1.0)** | **4.8 (1.7)** | **0.0135** |
| IFN-gamma | 7.8 (1.5) | 9.4 (5.7) | 0.4605 |
| IL-10 | 5.0 (2.6) | 9.1 (6.1) | 0.1170 |
| IL-13 | 3.2 (2.2) | 4.5 (3.3) | 0.3926 |
| IL-15 | 5.7 (1.5) | 7.6 (2.2) | 0.0913 |
| **IL-17A** | **2.9 (0.4)** | **7.1 (1.7)** | **0.0202** |
| IL-18 | 8.0 (1.6) | 9.2 (4.3) | 0.5637 |
| IL-1alpha | 6.5 (2.0) | 7.8 (2.3) | 0.2513 |
| IL-1beta | 9.7 (3.2) | 9.5 (4.4) | 0.9177 |
| IL-1RA | 18.7 (1.6) | 18.3 (3.1) | 0.7710 |
| **IL-27** | **9.8 (2.1)** | **12.6 (2.0)** | **0.0435** |
| IL-4 | 5.8 (0.5) | 8.3 (2.9) | 0.3138 |
| IL-6 | 13.0 (2.4) | 13.3 (3.2) | 0.7958 |
| IL-7 | 6.9 (1.7) | 6.5 (1.2) | 0.6502 |
| IL-8 | 13.5 (3.5) | 15.6 (3.0) | 0.2228 |
| **IL-12p70** | **3.7 (1.7)** | **7.3 (3.3)** | **0.0303** |
| IP-10 | 13.8 (2.1) | 15.3 (1.1) | 0.1116 |
| LIF | 9.3 (1.8) | 10.7 (4.3) | 0.4139 |
| MCP-1 | 13.6 (2.6) | 14.6 (2.8) | 0.4821 |
| MIP-1alpha | 10.1 (1.9) | 11.1 (3.7) | 0.6147 |
| MIP-1beta | 13.5 (2.1) | 14.2 (3.1) | 0.6932 |
| PDGFBB | 8.7 (1.8) | 7.7 (3.8) | 0.5362 |
| PIGF-1 | 7.1 (2.1) | 8.7 (1.8) | 0.1382 |
| RANTES | 9.1 (1.7) | 10.2 (2.5) | 0.3315 |
| SCF | 6.5 (1.8) | 7.7 (2.3) | 0.3102 |
| SDF-1a | 13.8 (2.1) | 15.8 (2.1) | 0.1063 |
| TNF-alpha | 8.1 (2.3) | 13.9 (5.5) | 0.0771 |
| VEGF-A | 16.9 (1.7) | 17.2 (1.7) | 0.6904 |
| **VEGF-D** | **6.3 (1.7)** | **9.9 (1.9)** | **0.0294** |

PARDS – pediatric acute respiratory distress syndrome, SD – standard deviation

**Supplementary Table 5B: Plasma cytokine concentration in severe and non-severe PARDS at timepoint 2**

| Cytokine concentration, pg/ml (log2) | Non-severe PARDS (n=8) | Severe PARDS (n=8) | T Test |
| --- | --- | --- | --- |
|  | Mean (SD) | Mean (SD) |  |
| **BDNF** | **7.2 (1.3)** | **4.5 (1.9)** | **0.0099** |
| BNGF | 8.4 (1.2) | 7.2 (2.1) | 0.3317 |
| EGF | 3.2 (1.3) | 3.2 (1.0) | 0.9050 |
| Eotaxin | 6.6 (1.0) | 7.1 (1.8) | 0.5686 |
| GM-CSF | 5.5 (1.8) | 5.8 (1.6) | 0.8004 |
| HGF | 9.2 (0.9) | 10.3 (1.0) | 0.0978 |
| IFN-alpha | 2.9 (1.3) | 2.1 (1.4) | 0.4075 |
| IFN-gamma | 7.8 (1.0) | 8.1 (2.1) | 0.7315 |
| IL-10 | 3.5 (0.6) | 3.2 (4.6) | 0.8531 |
| IL-13 | 2.1 (1.0) | 2.5 (1.6) | 0.6065 |
| IL-15 | 5.1 (2.3) | 5.5 (1.7) | 0.7297 |
| IL-17A | 4.2 (1.5) | 4.3 (1.8) | 0.9686 |
| IL-18 | 7.5 (1.0) | 8.0 (2.3) | 0.6234 |
| IL-1alpha | 0.0 (1.9) | 0.9 (2.7) | 0.5940 |
| IL-1beta | 1.5 (1.3) | 1.2 (2.3) | 0.7727 |
| IL-1RA | 9.5 (2.9) | 11.1 (3.3) | 0.3665 |
| IL-27 | 9.5 (2.4) | 10.0 (1.6) | 0.6964 |
| IL-4 | 4.0 (2.2) | 5.9 (1.5) | 0.1446 |
| IL-6 | 6.2 (2.6) | 5.5 (2.1) | 0.6702 |
| IL-7 | 1.6 (1.9) | 1.7 (1.7) | 0.8683 |
| IL-8 | 5.2 (1.3) | 3.5 (2.3) | 0.2354 |
| IL-12p70 | 2.9 (1.1) | 3.4 (2.3) | 0.6198 |
| IP-10 | 9.8 (1.7) | 10.3 (3.0) | 0.6961 |
| LIF | 4.1 (2.3) | 5.3 (1.4) | 0.3270 |
| MCP-1 | 7.4 (1.0) | 7.3 (1.0) | 0.7760 |
| MIP-1alpha | 4.8 (0.7) | 5.1 (1.6) | 0.6908 |
| MIP-1beta | 7.7 (0.5) | 7.7 (1.4) | 0.9278 |
| PDGFBB | 9.0 (0.6) | 8.9 (1.4) | 0.9230 |
| PIGF-1 | 3.4 (1.5) | 2.4 (1.3) | 0.2140 |
| RANTES | 7.6 (0.7) | 6.3 (1.6) | 0.3356 |
| SCF | 4.4 (1.3) | 5.6 (1.4) | 0.1195 |
| SDF-1a | 10.3 (0.4) | 9.9 (0.3) | 0.0988 |
| TNF-alpha | 3.3 (1.9) | 5.2 (2.1) | 0.1601 |
| VEGF-A | 9.6 (0.7) | 10.1 (2.5) | 0.6049 |
| VEGF-D | 5.4 (2.5) | 6.2 (2.2) | 0.5797 |

PARDS – pediatric acute respiratory distress syndrome, SD – standard deviation

**Supplementary Table 6: Overall cytokine concentrations in deep tracheal lavage and plasma in PARDS**

| Cytokine concentration, pg/ml (log2) | Deep tracheal lavage | Plasma | T test |
| --- | --- | --- | --- |
|  | Mean (SD) | Mean (SD) |  |
| BDNF | 6.8 (1.7) | 5.8 (1.9) | 0.0323 |
| BNGF | 10.7 (1.9) | 7.7 (1.6) | <0.0001 |
| EGF | 13.6 (2.3) | 3.4 (1.9) | <0.0001 |
| Eotaxin | 10.7 (2.3) | 6.7 (1.4) | <0.0001 |
| GM-CSF | 1.0 (2.8) | 5.0 (2.2) | 0.0091 |
| HGF | 14.5 (2.7) | 10.1 (1.3) | <0.0001 |
| IFN-alpha | 3.7 (2.5) | 1.9 (2.1) | 0.0155 |
| IFN-gamma | 8.8 (3.9 ) | 8.3 (2.5) | 0.5171 |
| IL-10 | 7.3 (3.5) | 3.3 (2.9) | 0.0001 |
| IL-13 | 3.9 (2.8) | 2.4 (1.2) | 0.0068 |
| IL-15 | 7.1 (2.7) | 5.3 (2.0) | 0.0105 |
| IL-17A | 6.2 (3.0) | 4.1 (2.1) | 0.0177 |
| IL-18 | 8.6 (2.8) | 7.9 (1.5) | 0.2851 |
| IL-1alpha | 6.9 (2.1) | 0.6 (2.7) | <0.0001 |
| IL-1beta | 8.7 (3.9) | 1.5 (1.7) | <0.0001 |
| IL-1RA | 18.5 (2.4) | 11.1 (3.0) | <0.0001 |
| IL-27 | 11.1 (2.5) | 9.5 (2.2) | 0.0208 |
| IL-4 | 6.2 (3.1) | 4.5 (2.2) | 0.0440 |
| IL-6 | 13.4 (2.8) | 6.4 (2.6) | <0.0001 |
| IL-7 | 6.8 (1.4) | 1.9 (1.3) | <0.0001 |
| IL-8 | 14.6 (3.8) | 7.9 (1.5) | <0.0001 |
| IL-12p70 | 5.5 (2.9) | 3.4 (1.5) | 0.0015 |
| IP-10 | 14.1 (1.8) | 10.5 (2.4) | <0.0001 |
| LIF | 10.4 (2.9) | 4.7 (2.1) | <0.0001 |
| MCP-1 | 13.5 (3.2) | 7.1 (1.0) | <0.0001 |
| MIP-1alpha | 10.1 (2.8) | 5.0 (1.1) | <0.0001 |
| MIP-1beta | 13.7 (2.7) | 7.8 (1.0) | <0.0001 |
| PDGFBB | 9.2 (2.7) | 8.8 (1.2) | 0.4532 |
| PIGF-1 | 7.2 (1.8 ) | 3.1 (1.2) | <0.0001 |
| RANTES | 9.5 (2.1) | 7.2 (1.4) | 0.0006 |
| SCF | 7.2 (2.2) | 4.9 (1.3) | <0.0001 |
| SDF-1a | 14.6 (2.1) | 10.2 (0.6) | <0.0001 |
| TNF-alpha | 9.5 (4.0) | 4.1 (2.0) | <0.0001 |
| VEGF-A | 16.4 (1.9) | 9.9 (1.7) | <0.0001 |
| VEGF-D | 7.6 (2.8) | 5.4 (2.3) | 0.0073 |

PARDS – pediatric acute respiratory distress syndrome, SD – standard deviation

**Supplementary Figure 1: Correlation between acute phase plasma cytokines and the oxygenation index**

*r*=Pearson’s correlation coefficient
